# A Clinicopathological Study of the Correlation Between H. pylori and Dyspepsia: A Cross-Sectional Study

**DOI:** 10.7759/cureus.90449

**Published:** 2025-08-18

**Authors:** Sushant Nair, Nitin Wasnik, Sagarika S Bhole

**Affiliations:** 1 General Surgery, NKP Salve Institute of Medical Sciences, Nagpur, IND

**Keywords:** dyspepsia, gastritis, helicobacter pylori, peptic ulcer, rapid urease test

## Abstract

Introduction

Dyspepsia is a common gastrointestinal (GI) complaint affecting a significant portion of the Indian population and is often associated with *Helicobacter pylori* infection. The clinical presentation alone may be insufficient to predict infection, necessitating a more comprehensive diagnostic approach. This study aimed to evaluate the association between *H. pylori* and dyspepsia, and to assess the related gastroduodenal pathologies using endoscopic and histopathological evaluations.

Methods

This cross-sectional study included 92 adult patients presenting with clinical symptoms of dyspepsia at a tertiary care hospital in central India from November 2022 to January 2024. All patients underwent upper gastrointestinal endoscopy and rapid urease test (RUT) for *H. pylori* detection. Biopsies were sent for histopathological analysis. Clinical, endoscopic, and histopathological findings were analyzed and compared between *H. pylori*-positive and *H. pylori*-negative groups using appropriate statistical tests.

Results

*Helicobacter pylori* infection was detected in 44 (47.8%) patients by RUT. Epigastric pain (76.1%) was the most prevalent symptom, although no significant association was found between clinical symptoms and *H. pylori* status (p > 0.05). Gastritis (72.8%) was the most common endoscopic finding, with a significant association between gastric ulcer and *H. pylori* positivity (p = 0.0368). Histopathological examination showed chronic inflammation in 12% of RUT-positive patients, which was statistically significant (p = 0.0346). Ultrasonographic findings did not show any significant correlation with *H. pylori* infection.

Conclusion

This study highlights the limited diagnostic value of symptoms alone in identifying *H. pylori* infection among dyspeptic patients. The findings support the routine use of endoscopic and histopathological evaluation in dyspepsia workup. Region-specific data such as this can aid in formulating more effective and individualized diagnostic and therapeutic strategies for dyspepsia management in Indian clinical settings.

## Introduction

Dyspepsia is a prevalent gastrointestinal (GI) disorder characterized by upper abdominal pain or discomfort. It encompasses a range of symptoms, including bloating, belching, early satiety, and nausea. Broadly, dyspepsia is classified into two categories: organic dyspepsia, linked to identifiable causes such as peptic ulcer disease, gastroesophageal reflux, or malignancy, and functional dyspepsia, in which no structural abnormalities are detected despite comprehensive evaluation [1,2].

Globally, the prevalence of dyspepsia varies from 10% to 40%, influenced by diagnostic criteria, population characteristics, and lifestyle factors. Dyspepsia represents a significant public health burden, leading to increased healthcare utilization and productivity loss [3]. In India, an estimated 30%-40% of the population may experience dyspeptic symptoms, with contributing factors such as dietary habits, socioeconomic status, genetic predisposition, and environmental exposures, most notably *Helicobacter pylori* infection [4,5].

*Helicobacter pylori* is a Gram-negative, spiral-shaped bacterium that colonizes the gastric mucosa and plays a well-established role in the pathogenesis of peptic ulcer disease, gastric carcinoma, and mucosa-associated lymphoid tissue (MALT) lymphoma [6]. Since its discovery in the early 1980s, *H. pylori* has fundamentally altered the diagnostic and therapeutic approach to upper gastrointestinal diseases [7]. India has one of the highest global burdens of *H. pylori* infection, with prevalence rates ranging between 50% and 80%, largely driven by poor sanitation, overcrowding, and fecal-oral transmission [8,9]. Most infections begin in childhood and may persist into adulthood without treatment, increasing the likelihood of chronic gastritis and dyspepsia.

Despite extensive research, the exact role of *H. pylori* in the etiology of dyspepsia, particularly functional dyspepsia, remains uncertain. Many patients infected with *H. pylori* do not experience symptom relief even after successful eradication therapy, suggesting that other pathogenic or host factors are at play [10-12]. Histological examination of gastric mucosa via biopsy remains essential for identifying chronic inflammatory changes, atrophy, or other histopathological alterations that may not be evident through endoscopy alone [13,14].

However, much of the clinical guidance on *H. pylori* testing and management is derived from Western populations [15], where disease prevalence, risk factors, and healthcare infrastructure differ markedly from the Indian context. There is growing recognition that extrapolating such data to Indian populations may lead to suboptimal or inappropriate management strategies.

Justification for the study

Although similar studies have been previously published, most are either outdated, geographically limited, or conducted in tertiary care centers with specific referral patterns that may not represent the broader population. Additionally, diagnostic modalities, clinical presentations, and treatment responses can vary significantly based on regional and socioeconomic factors. Hence, re-evaluating the association between *H. pylori* and dyspepsia in a representative Indian cohort remains essential.

This study aims to address these gaps by offering updated, region-specific data from central India, using both endoscopic and histopathological tools. By correlating clinical symptoms with diagnostic findings, it seeks to provide actionable insights into the management of dyspepsia and inform the development of more localized, evidence-based clinical guidelines.

Objectives of the study

Given this context, the present study was conducted with two primary objectives: to evaluate the association between *H. pylori *infection and dyspepsia in patients attending a tertiary care hospital in central India and to assess the spectrum of gastroduodenal pathologies in dyspeptic patients with confirmed *H. pylori* infection using endoscopic and histopathological analysis.

## Materials and methods

Study design and setting

This cross-sectional study was conducted in the department of surgery at a tertiary care hospital affiliated with a medical college in central India. The study period extended from November 2022 to January 2024.

Study population

The study population comprised adult patients (≥18 years) clinically diagnosed with dyspepsia. Dyspepsia was defined by the presence of one or more of the following symptoms: postprandial fullness, early satiety, epigastric pain, or epigastric burning sensation.

Inclusion criteria

We included patients aged ≥18 years and clinically diagnosed cases of dyspepsia.

Exclusion criteria

Patients below 18 years of age, patients who did not provide informed consent, and patients unwilling to undergo upper gastrointestinal (GI) endoscopy were excluded from this study.

Sample size estimation

The sample size was calculated using the following formula: N = 4P (100-P) / L.L, where P = 36% (prevalence of *H. pylori* in dyspeptic patients as per previous literature), L = 10 (absolute precision), and N = required sample size.

Based on the above parameters, the calculated minimum sample size was 92.

Sampling technique

A convenience sampling method was employed. All patients meeting the inclusion criteria during the study period were recruited consecutively, as the study was time-bound.

Data collection procedure

Following approval from the Institutional Ethics Committee of NKP Salve Institute of Medical Sciences and Research Center, Nagpur (approval number: 113/2022) and after obtaining written informed consent, eligible participants were enrolled. Data were collected using a structured, pre-designed case record form (CRF), which included relevant clinical history, physical examination findings, and investigation results.

Measurements and investigations

All patients underwent upper GI endoscopy. Biopsies were obtained where clinically indicated. Histopathological examination was conducted to detect *Helicobacter pylori* and to evaluate underlying gastric pathology.

Data management and confidentiality

Patient identifiers (e.g., name and contact details) were securely maintained by the principal investigator in a confidential file. Data were entered into Microsoft Excel (Microsoft Corp., Redmond, WA) and cross-verified on a weekly basis. Confidentiality was strictly maintained throughout the study, and datasets were routinely backed up and audited for accuracy.

Statistical analysis

Statistical analysis was performed using Epi Info version 7 (Centers for Disease Control and Prevention, Atlanta, GA). Descriptive statistics, including mean and standard deviation (SD), were used for continuous variables. Categorical variables were expressed as frequencies and percentages. Results were presented using appropriately formatted tables to enhance clarity and comprehension.

## Results

Rapid urease test (RUT) results

Out of 92 patients with dyspepsia, 44 (47.8%) tested positive for *Helicobacter pylori* by rapid urease test (RUT), while 48 (52.2%) were negative (Table 1).

**Table 1:** Distribution of RUT results (N = 92) RUT: rapid urease test

RUT result	Number	%
Positive	44	47.8%
Negative	48	52.2%

Clinical symptom distribution

The most common presenting symptom was epigastric pain (76.1%), followed by bloating (60.9%), aggravation on meals (64.1%), early satiety (53.3%), and belching (50%). Although a higher proportion of RUT-positive patients exhibited these symptoms, none showed statistical significance (p > 0.05) (Table 2).

**Table 2:** Distribution of clinical symptoms (N = 92) RUT: rapid urease test

Clinical symptoms	RUT positive (n = 44)	RUT negative (n = 48)	Total (N = 92)	p-value
Epigastric pain				
Present	31 (33.7%)	39 (42.4%)	70 (76.08%)	0.2504
Absent	13 (14.1%)	9 (9.8%)	22 (23.91%)	
Bloating				
Present	27 (29.3%)	29 (31.5%)	56 (60.86%)	0.3512
Absent	17 (18.5%)	19 (20.7%)	36 (39.13%)	
Aggravation on meals				
Present	27 (29.3%)	32 (34.8%)	59 (64.13%)	0.1789
Absent	17 (18.5%)	16 (17.4%)	33 (35.86%)	
Belching				
Present	22 (23.9%)	24 (26.1%)	46 (50%)	0.1156
Absent	22 (23.9%)	24 (26.1%)	46 (50%)	
Early satiety				
Present	24 (26.08%)	25 (27.17%)	49 (53.26%)	0.2820
Absent	20 (22.22%)	23 (25%)	43 (46.73%)	

Endoscopic findings

**Figure 1:**
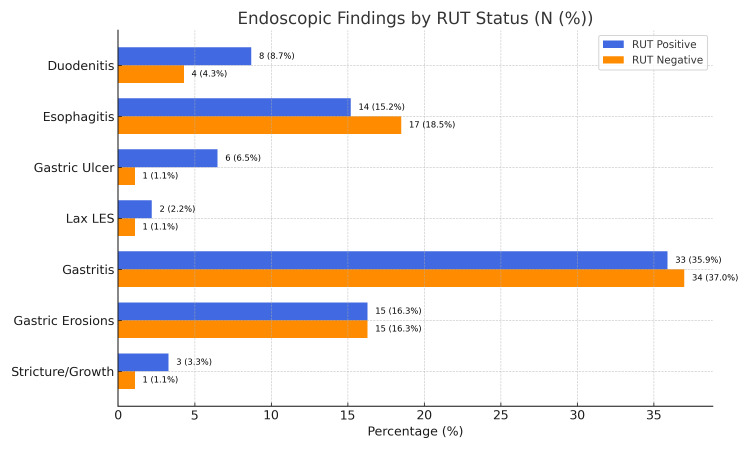
Endoscopic findings by RUT status Data presented as numbers (%) RUT: rapid urease test, LES: lower esophageal sphincter

The most common endoscopic finding was gastritis, observed in 67 (72.8%) patients, of whom 33 were positive for *Helicobacter pylori* on rapid urease test (RUT). Other notable findings included esophagitis in 31 (33.7%) patients, gastric erosions in 30 (32.6%) patients, duodenitis in 12 (13%) patients, gastric ulcers in seven (7.6%) patients, lax lower esophageal sphincter (LES) in three (3.3%) patients, and stricture or suspicious growth in four (4.3%) patients. Among these, the presence of gastric ulcer showed a statistically significant association with RUT positivity (p < 0.05 ), while the other endoscopic findings did not demonstrate any significant correlation (Figure 1).

Histopathological findings

Among the 44 RUT-positive patients, 11 (12%) showed chronic inflammation, which was statistically significant compared to the RUT-negative group (p < 0.05). Mild inflammation was seen in one (1.1%) patient, while one RUT-negative patient was diagnosed with adenocarcinoma (Table 3).

**Table 3:** Histopathological findings in RUT-positive patients (n = 44) RUT: rapid urease test

Histopathology	Number	%
Chronic inflammation	11	12%
Mild inflammation	1	1.1%
Adenocarcinoma	1	1.1%
Unremarkable histopathology	31	70.45%

Ultrasonographic evaluation in the RUT-positive group revealed that 23 (52.3%) patients had normal findings. Fatty liver was observed in five (11.4%) patients, while gastric outlet obstruction was noted in three (6.8%) patients. Additionally, one (2.3%) patient each demonstrated bilateral hydronephrosis and hepatomegaly. However, none of these ultrasonographic findings showed a statistically significant association with RUT positivity (p > 0.05) (Table 4).

**Table 4:** USG findings (N = 92) USG: ultrasonography, RUT: rapid urease test

USG	RUT	Number	%	p-value
Within normal limits	Positive	23	25%	0.4054
	Negative	26	28.3%	
Fatty liver	Positive	5	5.4%	0.2891
	Negative	4	4.3%	
Gastric outlet obstruction	Positive	3	3.3%	0.4620
	Negative	0	0%	
Pancreatitis	Positive	0	0%	0.5460
	Negative	4	4.3%	
Bilateral hydronephrosis	Positive	1	1.1%	1.00
	Negative	1	1.1%	
Cystitis	Positive	3	3.3%	1.00
	Negative	3	3.3%	
Liver parenchymal disease	Positive	0	0%	0.3492
	Negative	2	2.2%	
Hepatomegaly	Positive	2	2.2%	1.00
	Negative	2	2.2%	
Borderline prostatomegaly	Positive	3	3.3%	0.2894
	Negative	1	1.1%	
Bilateral echogenic kidneys	Positive	2	2.2%	1.00
	Negative	2	2.2%	
Colitis	Positive	0	0%	0.2687
	Negative	1	1.1%	
Altered liver echo pattern	Positive	2	2.2%	0.4125
	Negative	1	1.1%	
Liver hemangioma	Positive	0	0%	0.1104
	Negative	1	1.1%	

## Discussion

The present study identified *Helicobacter pylori* infection in 47.8% of dyspeptic patients, closely aligning with the prevalence reported by Akeel et al. [16] of 46.5% but lower than those reported by Agarwal et al. [17] of 76% and Yadav et al. [18] of 78%. This variability may be attributed to regional differences in socioeconomic conditions, dietary practices, sanitation standards, and diagnostic techniques.

The most affected age group in this cohort was 38-47 years, with a male predominance (63%), comparable to the findings from the study by Agarwal et al. [17] and Yadav et al. [18]. In contrast, studies by KC et al. [19] and Tanni et al. [20] reported a more balanced or female-dominant demographic, which may reflect variations in healthcare-seeking behavior and access across different regions.

Epigastric pain was the most commonly reported symptom (76%), followed by bloating, early satiety, belching, and meal-related symptom aggravation. While these findings are consistent with previously reported symptom patterns [17,18,21], no statistically significant association was observed between specific symptoms and* H. pylori* infection. This reinforces the established limitation of symptomatology as a standalone diagnostic tool for *H. pylori* detection.

Endoscopic examination revealed gastritis (72.8%) and gastric erosions (32.6%) as the most frequent findings. Notably, the presence of a gastric ulcer was significantly associated with *H. pylori* positivity (p = 0.0368), supporting similar observations by Yadav et al. [18] and KC et al. [19]. Conversely, other studies, such as those by Tanni et al. [20] and Kumari et al. [22], reported more heterogeneous endoscopic profiles, suggesting that while some findings may correlate with infection, endoscopic appearance alone lacks sufficient predictive value.

Histopathological analysis demonstrated a significant association between *H. pylori* infection and chronic gastritis (p = 0.0346), corroborating findings by Akeel et al. [16], Ahmed et al. [21], and Soomro et al. [23]. These results emphasize the diagnostic value of biopsy-based histological assessment, particularly in patients with nonspecific endoscopic presentations.

Ultrasonography yielded predominantly normal findings among *H. pylori*-positive patients (52%), with fatty liver (11%) and gastric outlet obstruction (6.8%) observed less frequently. No significant correlation was found between USG findings and *H. pylori* status, consistent with the limited diagnostic relevance of ultrasonography in dyspeptic evaluation, as noted by Gómez Zuleta et al. [24].

Overall, the findings of this study support a comprehensive diagnostic approach integrating clinical evaluation, endoscopy, and histopathology. While *H. pylori* remains an important etiological factor in dyspepsia, its presence does not uniformly explain symptomatology or disease severity. Context-specific, evidence-based strategies are essential for effective diagnosis and management, particularly in resource-limited settings.

## Conclusions

This study highlights the complex relationship between *Helicobacter pylori* infection and dyspepsia in the Indian clinical setting. Dyspepsia remains a multifactorial condition, and symptom-based diagnosis alone is insufficient. Our findings emphasize the value of a comprehensive diagnostic approach, integrating clinical assessment, endoscopy, and histopathology, to guide more accurate and individualized care.

Although *H. pylori *plays a significant role in upper gastrointestinal pathology, it is not the sole contributor to dyspeptic symptoms. Therefore, region-specific data should inform targeted investigations and treatment strategies, particularly in resource-constrained environments. These insights support the need for incorporating local evidence into national dyspepsia management guidelines.
